# Iron Depletion Affects Genes Encoding Mitochondrial Electron Transport Chain and Genes of Non­Oxidative Metabolism, Pyruvate Kinase and Lactate Dehydrogenase, in Primary Human Cardiac Myocytes Cultured upon Mechanical Stretch

**DOI:** 10.3390/cells7100175

**Published:** 2018-10-20

**Authors:** Magdalena Dziegala, Kamil A. Kobak, Monika Kasztura, Jacek Bania, Krystian Josiak, Waldemar Banasiak, Piotr Ponikowski, Ewa A. Jankowska

**Affiliations:** 1 Laboratory for Applied Research on Cardiovascular System, Department of Heart Diseases, Wroclaw Medical University, 50-367 Wroclaw, Poland; magdalena.dziegala@gmail.com (M.D.); kamkobak@gmail.com (K.A.K.); monikakasztura@gmail.com (M.K.); 2 Department of Food Hygiene and Consumer Health Protection, Wroclaw University of Environmental and Life Sciences, 50-375 Wroclaw, Poland; jacek.bania@up.wroc.pl; 3 Department of Heart Diseases, Wroclaw Medical University, 50-367 Wroclaw, Poland; krystian85@hotmail.com (K.J.); piotrponikowski@4wsk.pl (P.P.); 4 Centre for Heart Diseases, Military Hospital, Wroclaw, Poland; banasiak@4wsk.pl

**Keywords:** mitochondrial complexes, oxidative metabolism, non-oxidative metabolism, iron deficiency, iron excess, human cardiomyocytes

## Abstract

(1) Background: Oxidative energy metabolism is presumed to rely on the optimal iron supply. Primary human cardiac myocytes (HCM) exposed to different iron availability conditions during mechanical stretch are anticipated to demonstrate expression changes of genes involved in aerobic and anaerobic metabolic pathways. (2) Methods: HCM were cultured for 48 h either in static conditions and upon mechanical stretch at the optimal versus reduced versus increased iron concentrations. We analyzed the expression of pyruvate kinase (PKM2), lactate dehydrogenase A (LDHA), and mitochondrial complexes I–V at the mRNA and protein levels. The concentration of l-lactate was assessed by means of lactate oxidase method-based kit. (3) Results: Reduced iron concentrations during mechanical work caused a decreased expression of complexes I–V (all *p* < 0.05). The expression of PKM2 and LDHA, as well as the medium concentration of l-lactate, was increased in these conditions (both *p* < 0.05). HCM exposed to the increased iron concentration during mechanical effort demonstrated a decreased expression of mitochondrial complexes (all *p* < 0.01); however, a decrement was smaller than in case of iron chelation (*p* < 0.05). The iron-enriched medium caused a decrease in expression of LDHA and did not influence the concentration of l-lactate. (4) Conclusions: During mechanical effort, the reduced iron availability enhances anaerobic glycolysis and extracellular lactate production, whilst decreasing mitochondrial aerobic pathway in HCM. Iron enrichment during mechanical effort may be protective in the context of intracellular protein machinery of non-oxidative metabolism with no effect on the extracellular lactate concentration.

## 1. Introduction

Myocardium consists of cells associated with potent oxidative capacity [1]. However, the amount of ATP stored in these cells is limited and sufficient for few beats only. Therefore, the intact performance of cardiomyocytes is inevitably linked to the efficient oxidative metabolism localized in mitochondria [2,3].

Iron is presumed to be fundamental to this metabolic pathway in the context of both efficient oxygen storage and an undisturbed functioning of mitochondrial enzymes [4,5]. Due to the existence of iron in two interchangeable oxidative states, it constitutes an indispensable co-factor for the sequential oxidation-reduction reactions which yield in oxidative production of ATP. It is worth mentioning that limitation of aerobic metabolism shifts the cardiac energy production towards less favorable anaerobic glycolysis, which results mostly in lactate production [6]. Data from clinical studies indicate that iron deficiency (ID) is associated with higher mortality rates in patients with chronic heart failure (HF) [7,8], while iron supplementation improves symptoms and exercise capacity in these patients [9,10].

Accordingly, we investigated the influence of different iron availability conditions introduced to primary human cardiac myocytes (HCM) cultured either in static conditions or upon mechanical stretch (which mimics physiological work of the cardiac cells) on the medium lactate concentration and the expression of genes involved in oxidative and non-oxidative metabolic pathways in these cells.

## 2. Materials and Methods

### 2.1. Cell Culture Conditions

Primary HCM (obtained from PromoCell, Heidelberg, Germany) were cultured in Myocyte Growth Medium supplemented with the recommended Supplemented Mix (obtained from PromoCell, Heidelberg, Germany) for 60 days in order to induce differentiation towards myotube-like structures and branch-like structures. Notably, the aforementioned duration of cell differentiation enabled realization of fully mature cardiac myocytes. For passaging, cells were treated with DetachKit (from PromoCell). The cells were maintained according to manufacturer’s protocol.

### 2.2. Experimental Schedule

**Figure 1:**
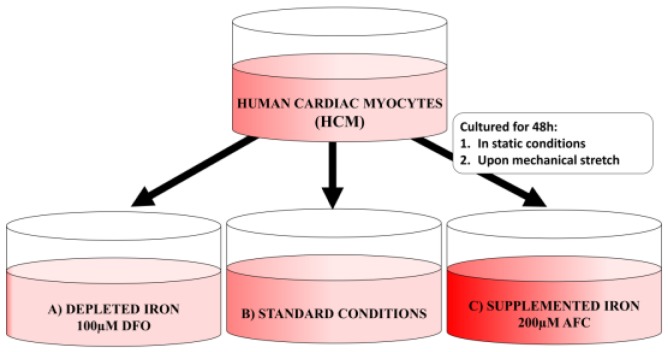
Schematic presentation of human cardiac myocyte (HCM) cell culture held either in static conditions or upon mechanical stretch and exposed to different iron concentrations: (**A**) decreased iron concentration (iron chelation with the use of 100 μM DFO); (**B**) optimal iron concentration (standard iron concentration in Myocyte Growth Medium with Supplement Mix); and (**C**) increased iron concentration (iron supplementation with the use of 200 μM AFC). DFO, deferroxamine; AFC, ammonium ferric citrate.

HCM were cultured for 48 h in static conditions (standard cell culture incubator) or upon mechanical stretch (with the use of FlexerCell Strain Unit placed in the standard cell culture incubator). In order to achieve different iron availability conditions, the cell culture medium was supplemented with 100 μM deferoxamine (DFO; Sigma-Aldrich; Merck KGaA, Darmstadt, Germany) or 200 μM ammonium ferric citrate (AFC; Sigma-Aldrich; Merck KGaA) (Figure 1). Specifically, for mimicking the physiological work of the cardiac cells, HCM were cultured on the type 1 collagen-coated 6-well plates and the mechanical stretch was introduced with the use of FlexerCell Strain Unit (Flexcell®, biaxal cyclic stretch (15%, 0.5 Hz)). Both DFO and AFC were chosen and applied based on available data from cell culture studies [11,12,13]. After being diluted by a factor of 1000, the compounds were added to the cells. 

### 2.3. Iron Content in Cells

The intracellular iron content was assessed by means of flame atomic absorption spectroscopy assay [14], as described in detail in our previous work [15].

### 2.4. Reverse Transcription-Quantitative Polymerase Chain Reaction (RT-QPCR)

Total RNA extraction was performed according to manufacturer’s instructions (RNeasy Mini Kit, Qiagen, Hilden, Germany). In order to remove genomic DNA, an on-column DNAse digestion was carried out. For the synthesis of first-strand cDNA, the SuperScript III First-Strand Synthesis System with oligo(dT)20 primer (Invitrogen, Carlsbad, CA, USA) was applied. The following primer sequences were used and designed with Beacon Designer Software (version 2.0, Bio-Rad Laboratories, Inc., Hercules, CA, USA): (1) genes of enzymes involved in non-oxidative metabolism, pyruvate kinase (PKM2) forward: 5′-AGTGGGGCCATAATCGTCCT-3′ and reverse: 5′-CACGCATGGTGTTGGTGAAG-3′; lactate dehydrogenase A (LDHA) forward: 5′-TTGTAAAATACAGCCCGAACTGCAA-3′ and reverse: 5′-CCCAGGATGTGTAGCCTTTGA-3′; (2) genes of mitochondrial enzymatic complexes involved in oxidative metabolism (respiratory chain), complex I (subunit NDUFS1) forward: 5′-ACCGAGCCAATGGTCAGAAA-3′ and reverse: 5′-CTGCAACATTCCAGCTACGC-3′; complex II (subunit 30kDa) forward: 5′-TCCTATGTGGACGTTGGCAC-3′ and reverse: 5′-GAAAGTTGGGCAGTAGGAGC-3′; complex III (subunit UQCRFS1) forward: 5′-AACCCCTGTTTGTGCGTCAT-3′ and reverse: 5′-TTGAGAGGAGCAGGACCCAA-3′; complex IV (subunit COX4I1) forward: 5′-ACGAGTGGAAGACGGTTGTG-3′ and reverse: 5′-AGGGGGCCGTACACATAGT-3′; complex V (subunit ATP5B) forward: 5′-AGACTGGTTTTGGAGGTGGC-3′ and reverse: 5′-GGCCAAAGTCTCAGGACCAA-3′. In order to prevent the amplification of genomic DNA, the primers were designed from specified exon–exon junctions of genes of interest. The selection of reference genes were carried out by means of geNorm analysis (geNorm kit, ge-SY-12; PrimerDesign, Ltd., Southampton, UK), as a result of which the combination of two reference gene, β-actin (actb; Homo sapiens) and 18S ribosomal RNA (rRNA; 18S; Homo sapiens), was identified as the most stable reference value across all experimental conditions. (All experiments were performed in triplicate and repeated three times independently. Real-time qPCR was carried out using CFX ConnectTM Real-Time PCR Detection system (Bio-Rad Laboratories, Inc., Hercules, CA, USA ) and SsoFastTM EvaGreen^®^ Supermix reagent (Bio-Rad Laboratories, Inc.). The assay procedure was comprised of an initial denaturation at 95 °C, followed by 40 cycles of denaturation steps at 95 °C for 10 s and primer annealing/extension at 60 °C for 15 s. For each reaction, a melt curve analysis was performed in order to determine the specificity of PCR. The amplification efficiency was estimated by running serial dilutions of a template. The standard curve was obtained by plotting successive dilutions against the relevant Cq values. Based on the slope of the standard curve, the amplification efficiency (E) was calculated using the equation: E = 10^−1^/slope. For the target amplicons, β-actin and 18S rRNA, the amplification efficiencies were not comparable and the relative expression was calculated using the Pfaffl method [16].

### 2.5. Western Blotting

HCM cells with the number of 2.5 × 10^5^ from each series of experiments were homogenized on ice in RIPA buffer (10 mM Tris-Cl (pH 8.0), 1 mM EDTA, 0.5 mM EGTA, 1% Triton X-100, 0.1% sodium deoxycholate, 0.1% SDS, 140 mM NaCl, 1 mM PMSF) for 60 min, followed by sonication on ice (20 kHz; 10 s). The protein concentration was estimated by BCA assay [17]. In order to determine protein levels of enzymes of non­oxidative metabolism and mitochondrial complexes, 30 μg of appropriate protein lysates (about 10 μL) were added on 12% SDS/polyacrylamide gel. Proteins were electro-transferred on PVDF membranes (Millipore, Billerica, MA, USA) in Towbin buffer (25 mM Tris, 192 mM glycine, 20% methanol) for LDH and PKM2 or in CAPS buffer (10 mM CAPS, pH 11, 10% methanol) for OXPHOS cocktail. The membranes were blocked with 5% skimmed milk for 1 h and incubated with primary antibodies (Table 1) overnight. After washing, membranes were incubated with secondary horseradish-conjugated anti-rabbit and anti-mouse antibodies (Table 1) and developed with the ECL or Femto detection system (Pierce Biotechnology, Rockford, IL, USA). The data were obtained from 3 separate experiments.

### 2.6. l-Lactate Concentration Measurement

HCM cell culture supernatants collected from each series of experiments were centrifuged at 15,800× *g* for 10 min and frozen in −80 °C for several weeks. The l-lactate concentration was estimated using the lactate oxidase method-based kit (Randox Laboratories, Crumlin, UK). The data were obtained in triplicate from 3 separate experiments.

### 2.7. Statistical Analysis

Unless otherwise indicated, data were depicted as the mean ± standard deviation. Differences in mean values between groups were analyzed with the Kruskal–Wallis test followed by post-hoc Dunn’s multiple comparison test. All experiments were performed in triplicate and repeated three times independently. *p* < 0.05 was used to indicate a statistically significant difference.

## 3. Results

### 3.1. Changes in Intracellular Iron due to the Addition of Iron Chelator, Deferroxamine (DFO), or Iron Salt, Ammonium Ferric Citrate (AFC), to Culture Media

The mean intracellular iron content in HCM cells cultured in the standard Myocyte Growth Medium was 1.08 ± 0.12 mg/L. The direct assessment of intracellular iron revealed that the addition of 100 μM DFO to the medium caused a decrease of intracellular iron concentration to 0.55 ± 0.08 mg/L. On the contrary, a treatment of HCM cells with 200 μM AFC increased the mean intracellular iron concentration to 5.43 ± 0.15 mg/L (data not shown).

### 3.2. Effects of Differing Iron Availability either in Static Conditions or upon Mechanical Stretch on the Expression of Genes of Mitochondrial Complexes in Primary Human Cardiac Myocytes

**Figure 2:**
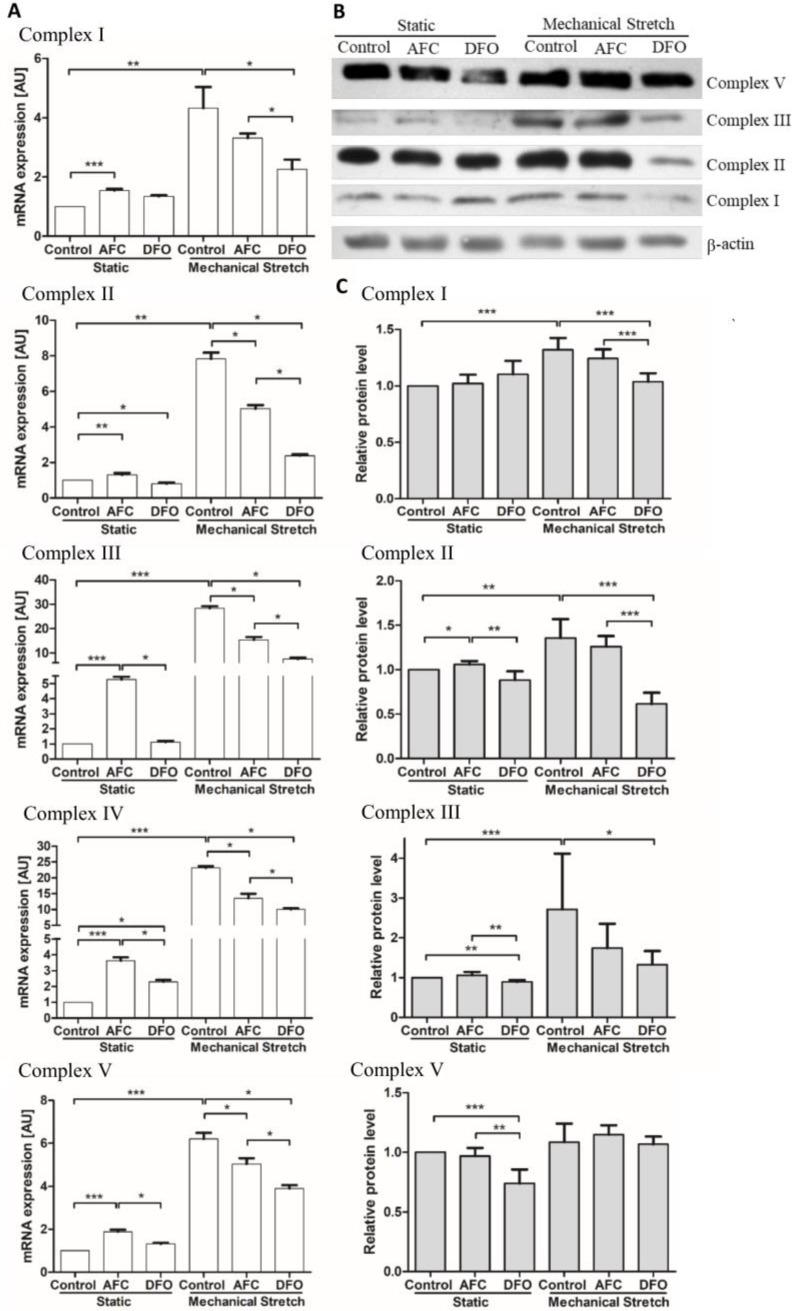
Expression of mitochondrial complexes in HCM cells cultured either in static conditions or upon mechanical stretch with concomitant optimal, increased or reduced iron availability. mRNA expression levels of complexes I, II, III, IV and V in HCM cells (**A**). Representative immunoblot of complexes I, II, III, V and β-actin as a loading control (**B**) and summary data showing relative protein expression in cell lysates standardized to β-actin (**C**). Complex IV was undetectable in the cell lysates. Data are presented as the mean + standard deviation obtained from three separate experiments. * *p* < 0.05; ** *p* < 0.01; *** *p* < 0.001. AU, arbitrary units; AFC, ammonium ferric citrate; DFO, deferroxamine.

The effects of iron availability on the levels of genes of mitochondrial complexes were assessed (Figure 2A–E). The iron-enriched environment introduced in the static conditions caused an increase in mRNA expression of all investigated mitochondrial complexes (all *p* < 0.0001). HCM cells exposed to mechanical stretch (that reflects cardiac beat conditions in the in vitro model) demonstrated, as compared to the cells cultured in static conditions, an increased mRNA expression of genes of mitochondrial complexes (all *p* < 0.05), suggesting an augmented energetic demand. Notably, the reduced iron concentration in the condition of mechanical work caused a decrease in mRNA levels of mitochondrial complex I, complex III, complex IV and complex V (all *p* < 0.05), indicating a limited oxidative metabolism. The exposure of the human cardiomyocytes to the increased iron concentration during mechanical effort also eventuated, as compared to the cells cultured upon the mechanical stretch and optimal iron concentration, in a decreased mRNA expression of mitochondrial complexes (all *p* < 0.01); however, a decrement was significantly smaller than in case of iron chelation (*p* < 0.05). Western blot analysis revealed similar patterns of changes at the protein level (Figure 2F).

### 3.3. Effects of Differing Iron Availability either in Static Conditions or upon Mechanical Stretch on the Expression of Genes of Non-Oxidative Metabolism, Pyruvate Kinase and Lactate Dehydrogenase, in Primary Human Cardiac Myocytes

**Figure 3:**
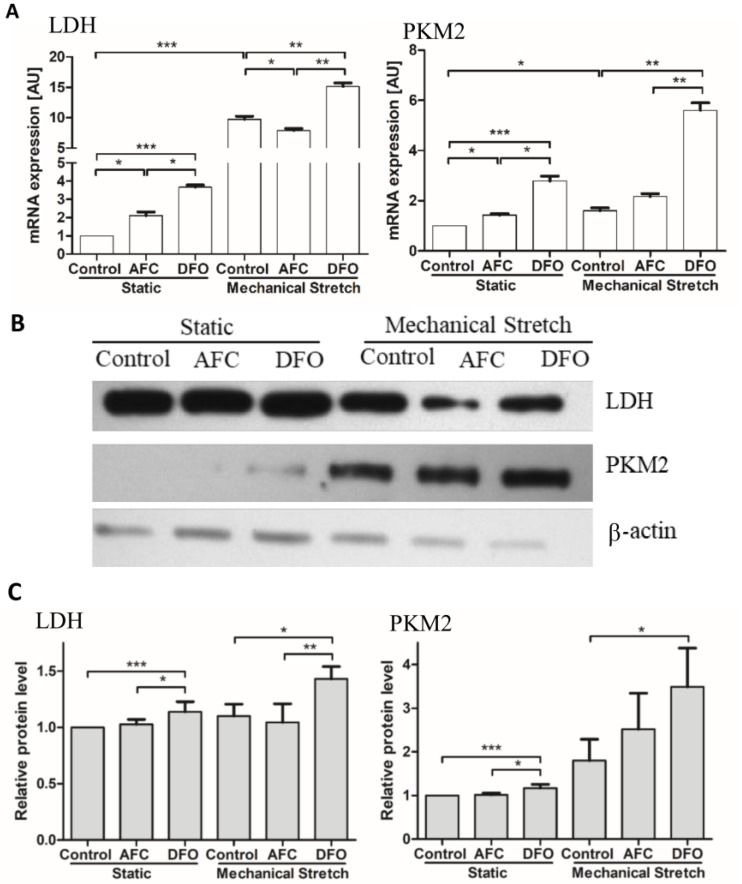
Expression of enzymes of non-oxidative metabolism in HCM cells cultured in static conditions or upon mechanical stretch with concomitant optimal, increased or reduced iron availability. mRNA expression levels of LDHA and PKM2: (**A**) representative immunoblot of LDH and PKM2 and β-actin as a loading control; (**B**) summary data showing relative protein expression in cell lysates standardized to β-actin. (**C**) Data are presented as the mean ± standard deviation obtained from three separate experiments. * *p* < 0.05; ** *p* < 0.01; *** *p* < 0.001. AU, arbitrary units; LDHA, lactate dehydrogenase A; PKM2, pyruvate kinase; LDH, lactate dehydrogenase; AFC, ammonium ferric citrate; DFO, deferroxamine.

We found that in static conditions HCM cells exposed to both decreased and elevated iron availability conditions demonstrated an increase in mRNA expression of genes of non-oxidative metabolism (all *p* < 0.05) (Figure 3A,B). Notably, an increase in mRNA levels of both investigated genes was higher in the cells treated with DFO as compared to HCM exposed to iron salt (both *p* < 0.05). In case of mechanical stretching of human cardiomyocytes, as compared to the cells cultured in static conditions, we observed an increased mRNA expression of genes of non-oxidative metabolism (both *p* < 0.05). Iron-deficient environment introduced during mechanical effort caused further increase in mRNA levels of PKM2 and lactate dehydrogenase (both *p* < 0.01). On the contrary, iron-enriched medium in the working conditions of HCM cells, as compared to the conditions of stretching and optimal iron availability, caused a decrease in mRNA expression of LDHA (*p* < 0.05) (Figure 3A). The analysis of the expression of respective genes at the protein level demonstrated similar pattern of changes (Figure 3C).

### 3.4. Effects of Differing Iron Availability in Static Conditions or upon Mechanical Stretch on the Concentration of l-Lactate Secreted to Culture Medium by Primary Human Cardiac Myocytes

**Figure 4:**
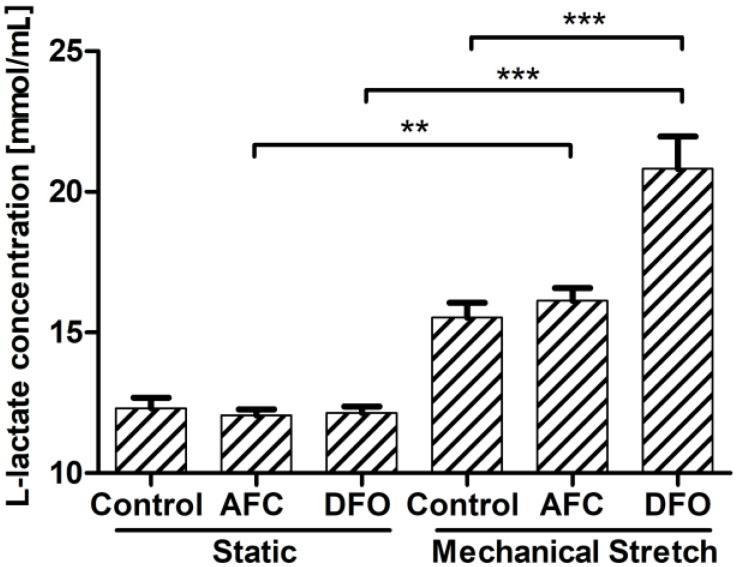
Concentration of extracellular l-lactate measured in medium of HCM cells cultured either in static conditions or upon mechanical stretch with concomitant optimal, increased or reduced iron availability conditions. ** *p* < 0.01; *** *p* < 0.001. AFC, ammonium ferric citrate; DFO, deferroxamine.

The exposure of HCM to increased or reduced iron availability in the static conditions did not influence the concentration of l-lactate produced to the culture medium (Figure 4). Further, HCM cultured in the conditions of mechanical effort, as compared to those cultured in static conditions, did not demonstrate a significant change in l-lactate secretion. Notably, iron-deficient environment during mechanical stretching, as compared to the conditions of the mechanical effort and optimal iron concentration, eventuated in an increased l-lactate concentration measured in the cell culture medium (*p* < 0.001). An addition of iron salt to the medium during mechanical stretching, as compared to the conditions of the mechanical effort and optimal iron concentration, did not influence the secretion of l-lactate by HCM cells (Figure 4).

## 4. Discussion

Our results describe for the first time the influence of both ID and iron supplementation in the conditions that mimic physiological work of the cardiac cells on genes involved in oxidative and non-oxidative metabolism in primary HCM. We showed that during mechanical stretching, reduced iron availability upregulates the genes of both glycolysis and lactate formation pathways, whilst decreasing the expression of key players of mitochondrial oxidative route. On the other hand, iron supplementation during mechanical effort downregulates a lactate dehydrogenase, an enzyme involved in lactate production, and may act in a protective manner in the context of limitation of cellular acidification.

It is worth noting that aerobic and non-aerobic metabolic pathways of cardiac myocytes have been insufficiently investigated in the context of iron metabolism. To date, there have been few studies that linked iron overload to low ATP production and decreased activity of mitochondrial complexes I–IV in rat heart mitochondria [18,19] or to reduced glucose oxidation in murine cardiac muscle [20]. More research attention has been given to ID-induced alterations of mitochondrial oxidative enzymatic machinery. Indeed, ID has been confirmed to lower the expression and activity of mitochondrial complexes and to upregulate the lactate dehydrogenase in cardiomyocytes from mice [21,22,23]. On the contrary, Melenovsky et al. reported the preserved activity of respiratory chain enzymes in iron-deficient myocardium derived from patients with HF [24]. However, one should bear in mind that cardiac tissue samples would in fact comprise, apart from cardiomyocytes, cardiac fibroblasts, endothelial cells, and vascular smooth muscle cells [25]. Instead, our study provides an insight into the metabolic response of isolated primary HCM to different iron availability conditions in the in vitro model of cardiac work.

With this work, we demonstrated that the influence of different iron concentrations on human cardiac cells is apparent even in static conditions of cell culture. Indeed, the increment in iron concentration leads to an increased expression of genes of oxidative metabolism in human cardiomyocytes. This finding suggests that augmented iron availability promotes protein machinery that governs oxidative energy production in cardiac cells. As regards the anaerobic pathway, both elevated and decreased iron availability conditions cause an upregulation of PKM2 and LDHA, but the increase in expression of these genes was significantly greater in case of iron chelator treatment.

In this study, we also presented evidence on work-induced increase in expression of genes of both aerobic and non-aerobic pathways in human cardiomyocytes. Furthermore, we reported a decrease of complexes I–V upon both iron chelation and iron supplementation during mechanical effort, which is in accordance with previously cited findings. Notably, our study showed that ID reduces the expression of mitochondrial complexes to a greater extent than iron supplementation. The present work extends the knowledge on the influence of iron availability on genes of non-oxidative metabolism. In particular, we demonstrated, during mechanical effort, not only an increase of both PKM2 and LDHA in iron-deficient conditions, but also a decrease of LDHA caused by iron supplementation. Further, our results indicated that extracellular lactate production and resultant acidification are significantly higher upon treatment with iron chelator, while an addition of iron salt does not influence the medium lactate concentration. These findings may serve as a valuable step in an understanding of beneficial effects of iron therapy reported in patients with chronic HF, including a reduction in the plasma level of NT-proBNP, improvement in echocardiographic measures of myocardial systolic and diastolic function, and amelioration of exercise capacity [26,27,28,29,30].

It is worth noting that our results are consistent with the study of Walter et al., who demonstrated that both iron overload and ID are detrimental to the functioning of liver mitochondria [31]. However, with this work, we showed that upon mechanical stretch, the decreased iron availability affects HCM more unfavorably in the context of both mitochondrial genes of energy metabolism and extracellular acidification.

In conclusion, we managed to demonstrate that, in working HCM, ID has a more negative impact on both the genes of oxidative metabolism and lactate production than increased iron availability. Moreover, the iron-enriched environment seems to have some protective properties for working cardiomyocytes illustrated by an influence on intracellular protein machinery of non-oxidative metabolism with no effect on the extracellular lactate concentration.

## Figures and Tables

**Table 1:** Antibodies and dilutions used for Western blotting.

Antigen	Dilution	Manufacturer	Ref. Number
LDH	1:1000	Santa Cruz Biotechnology	sc-33781
PKM2	1:750	Invitrogen	PA5-13980
Total OXPHOS cocktail ^1^	1:500	Abcam	ab110411
Beta-actin HRP	1:5000	Santa Cruz Biotechnology	sc-1616 HRP
Rabbit IgG HRP	1:40,000	Jackson ImmunoResearch	111-035-045
Mouse IgG HRP	1:40,000	Sigma-Aldrich ^2^	A 9917

^1^ Complex I (NDUFB8); complex II (subunit 30 kDa); complex III (subunit Core 2); complex IV (subunit II); complex V (subunit alpha); ^2^ company locations are as follows: Santa Cruz Biotechnology, Inc. (Dallas, TX, USA); Jackson ImmunoResearch Laboratories, Inc. (West Grove, PA, USA); Sigma-Aldrich (Merck KGaA). LDH, lactate dehydrogenase; PKM2, pyruvate kinase; OXPHOS, oxidative phosphorylation; HRP, horseradish peroxidise; IgG, immunoglobulin G.

## Abbreviations

HCM	human cardiac myocytes
PKM2	pyruvate kinase
LDHA	lactate dehydrogenase A
ATP	adenosine triphosphate
ID	iron deficiency
HF	heart failure
DFO	deferroxamine
AFC	ammonium ferric citrate
RIPA	radioimmunoprecipitation assay
RT-qPCR	reverse transcription-quantitative polymerase chain reaction
cDNA	complementary deoxyribonucleic acid
rRNA	ribosomal ribonuclein acid
BCA	bicinchoninic acid
OXPHOS	oxidative phosphorylation
